# Artificial intelligence–powered insights into high-risk, non-obstructive coronary atherosclerosis: a case report

**DOI:** 10.1093/ehjcr/ytae172

**Published:** 2024-04-09

**Authors:** Andrea Provera, Daniele Andreini, Kersten Petersen, Emanuele Gallinoro, Edoardo Conte

**Affiliations:** Department of Clinical and Biomedical Sciences, University of Milan, Milan, Italy; Department of Clinical and Biomedical Sciences, University of Milan, Milan, Italy; Department of Clinical Cardiology and Cardiovascular Imaging, IRCCS Ospedale Galeazzi Sant’Ambrogio, Via Cristina Belgioioso 173, Milan 20157, Italy; HeartFlow, Inc., Redwood City, CA, USA; Department of Clinical Cardiology and Cardiovascular Imaging, IRCCS Ospedale Galeazzi Sant’Ambrogio, Via Cristina Belgioioso 173, Milan 20157, Italy; Department of Clinical Cardiology and Cardiovascular Imaging, IRCCS Ospedale Galeazzi Sant’Ambrogio, Via Cristina Belgioioso 173, Milan 20157, Italy

**Keywords:** Case report, Plaque analysis, Artificial intelligence

## Abstract

**Background:**

Advanced coronary plaque analysis by cardiac computed tomography (CT) has recently emerged as a promising technique for better prognostic stratification. However, this evaluation application in clinical practice is still uncertain.

**Case summary:**

In the present case, we described the clinical picture of a 44-year-old tennis player with ectopic ventricular beats in which cardiac CT enabled the identification of a non-obstructive but high-risk plaque on proximal left anterior descendent artery. The application of artificial intelligence (AI)-enhanced software enabled to better stratify the patients’ risk. The present case describes how early identification of non-obstructive but high-risk coronary plaque evaluated by cardiac CT using AI-enhanced software enabled accurate and personalized risk assessment.

**Discussion:**

The main clinical message of this case report is that advanced plaque analysis by cardiac CT, especially when performed with AI-based software, may provide important prognostic information leading to a personalized preventive approach. Moreover, AI-based software may contribute to promote a routine evaluation of these important data already included in traditional cardiac CT.

**Learning points**

- Cardiac computed tomography may provide significant insight on coronary atherosclerosis subtypes beyond lumen stenosis
- Artificial intelligence–based software for advance plaque analysis may fasten and facilitate plaque analysis, enhancing its application clinical application on a regular basis.

## Introduction

Coronary artery disease (CAD) is the leading cause of cardiovascular mortality and morbidity in the adult population in Western countries; it is characterized by the presence of atherosclerosis in epicardial coronary arteries. US data show that in 2015, CAD accounted for 8.9 million deaths and 164.0 million disability-adjusted life years (DALYs).^1,2^

Several mechanisms result in atherosclerotic plaque, a dynamic lesion mainly composed of adipocytes and inflammatory cells affecting the epicardial coronary vessels leading to their obstruction. Over time, a plaque in a coronary wall can undergo sudden ulceration, becoming an unstable plaque due to the prevailing inflammatory component and thin fibrotic cap causing an acute coronary syndrome (ACS).^2^ Instead, the plaque with a thick fibrous cap and less inflammatory components develops a more stable condition causing symptoms of exercise angina only when vessel obstruction causes loss of coronary flow reserve (CFR).^2,3^

Over the last few decades, the use of a new generation of multidetector computed tomography (MDCT) scans and software enabled the assessment of the degree of vessel stenosis and also the composition^4–6^ of the plaque itself which are correlated with ACS.^7–9^

**Figure 1:**
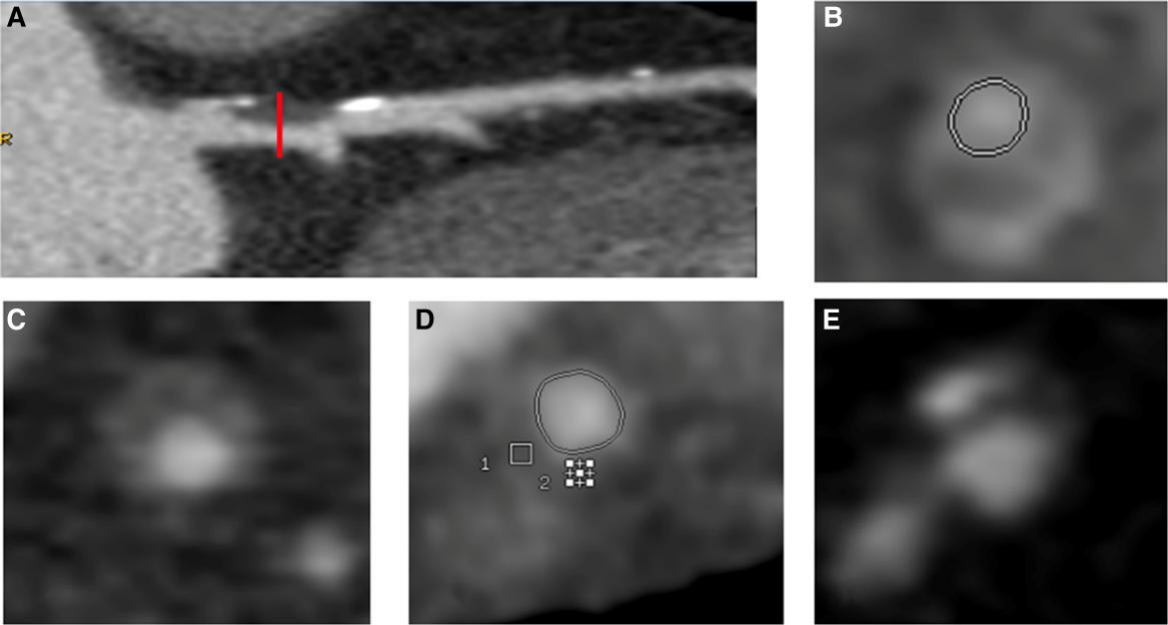
Computed tomography coronary angiography (CTCA) images of four ‘high-risk’ plaque phenotypes. (*A*) Positive remodelling: ratio of the outer vessel diameter of the plaque divided by outer diameter of the vessel, proximal and distal, >1.1. (*B*) Napkin ring sign: low-attenuation area adjacent to the coronary lumen, surrounded by a ring of higher attenuation (<130 HU). (*C* and *D*) Low attenuation: non-calcified plaque with internal attenuation < 30 HU.^8^ (*E*) Spotty calcification: plaque with punctate calcium.

Four plaque, so-called high-risk, phenotypes have been described: napkin ring sign, positive remodelling, spotty calcification, and low-attenuation plaque (*Figure 1*), which shared high inflammatory component and seem to be correlated with a greater predisposition to plaque rupture and subsequent occurrence of ACS.^6,7,9,10^ However, advance plaque evaluation by cardiac CT is often time-consuming and suffer from inter-observer variability. Artificial intelligence (AI)-based software may assist clinicians in assessing plaque features during clinical routine.

The present case highlights the potential role of advanced plaque evaluation in the early detection of high-risk coronary atherosclerosis with the potential aid of AI.

## Summary figure

Left side of the illustration (panel A): semi-automatic plaque analysis software (plaque ID analysis using AW server GE) shows on proximal left anterior descendent artery (LAD) low-attenuation pattern (<30 HU) and calcified plaque on mid-LAD. Right side of the illustration (panel B): artificial intelligence (AI)-based software (HeartFlow®) showing plaque analysis of the same segment of LAD detailing total plaque volume 337 mm^3^ of which 31 mm^3^ (9%) of calcified plaque, 306 mm^3^ (88%) of non-calcified plaque, and 10 mm^3^ (3%) of low-attenuation plaque. The semi-automatic tool provides similar data regarding total and subtype plaque volume, but AI-based is less time-consuming and is independent of the computed tomography (CT) readers’ experience.

**Figure:**
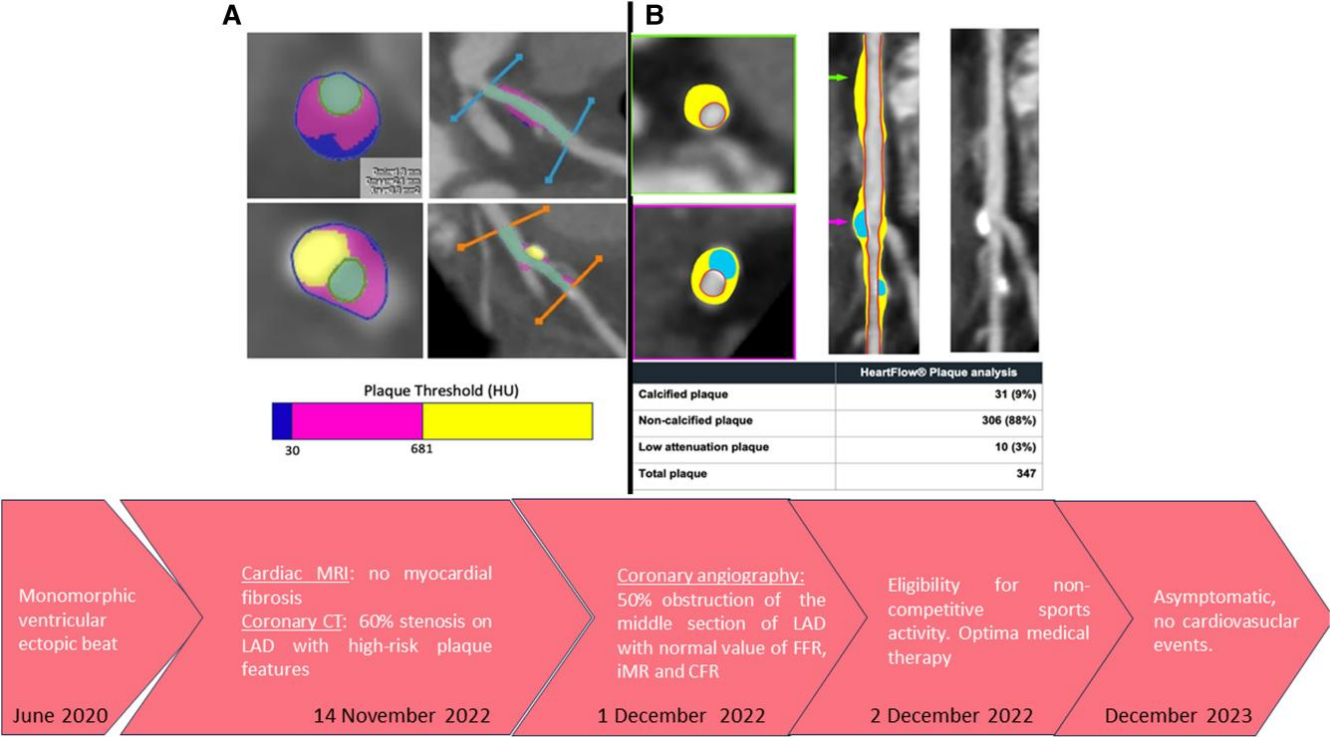


## Case summary

We present the case of a 44-year-old Caucasian male, master tennis player with low cardiovascular risk and no previous relevant medical history except for allergic asthma, presenting to our attention after the identification of monomorphic ventricular ectopic beats (VEBs) at rest [left bundle branch block (LBBB) morphology with intermediate axis] that were not abolished during cycle ergometer test. He was asymptomatic for palpitations and he was not receiving any medical treatment.

His father suffered from ischaemic heart disease, and no other relevant familiar cardiovascular history was reported.

On physical examination, blood pressure was 120/80 mmHg, and no murmurs, rubs, or gallops were present at cardiac auscultation. Peripheral pulses were normo-isosfigmic. No jugular turgor and no peripheral oedema were present. Electrocardiogram (EKG) was normal.

Laboratory tests showed normal values of blood count and coagulation parameters. Fasting blood glucose was 93 mg/dL; low-density lipoprotein (LDL)/high-density lipoprotein (HDL) was 153/52 mg/dL, respectively; serum creatinine level was 1.11 mg/dL; and plasma electrolytes were within normal limits. Inflammation marker [C-reactive protein (CRP)] was within normal ranges.

He subsequently performed a transthoracic echocardiography which showed normal biventricular systolic and diastolic function without valvular and pericardial disease.

A 24 h EKG monitoring was also performed which showed normal sinus rhythm interrupted by sporadic supraventricular ectopic beat and isolated but frequent ventricular beat (1220 VPB/24 h) suppressed during training. The patient was advised to undergo cardiac magnetic resonance (CMR) with gadolinium which showed no structural heart disease or myocardial fibrosis and cardiac computed tomography (CCT) which showed the presence of CAD in the middle section of the anterior descending artery, resulting in 60% stenosis. Moreover, fibroatheroma with high-risk plaque features and minimum lumen area (MLA) value of 3.3 mm^2^ was evident in the proximal segment of left anterior descendent artery (LAD) (Summary figure).

Of interest, advanced coronary plaque analysis was performed both by dedicated semi-automatic software, previously validated vs. intravascular ultrasound (IVUS),^11^ and by an AI-based software (HeartFlow®), which showed on proximal LAD total plaque volume of 337 mm^3^ of which 31 mm^3^ (9%) of calcified plaque, 306 mm^3^ (88%) of non-calcified plaque, and 10 mm^3^ (3%) of low-attenuation plaque as shown in the Summary figure. Interestingly, both AI and semi-automatic software provide similar data regarding total and subtype plaque volume, but AI-based software was faster (5 min for AI software vs. 30 min for semi-automatic software), less demanding for imaging cardiologists, and independent from the experience of CT reader.

Due to CT findings, the patient started therapy with atorvastatin 20 mg/die tablets and single-antiplatelet therapy (SAPT) with clopidogrel 75 mg/day tablets, due to a reported allergy to aspirin (ASA).

**Figure 2:**
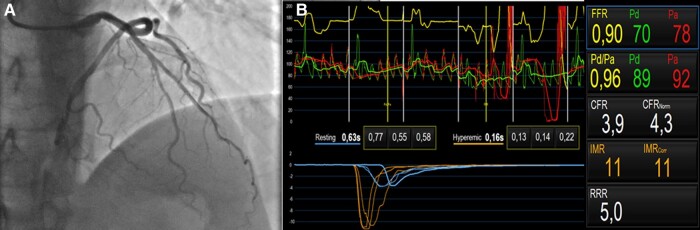
(*A*) Coronary angiography of left anterior descendent artery (LAD) which showed moderate stenosis (50%) on the middle section of LAD. (*B*) Functional physiology which showed normal values of fractional flow reserve (FFR) (0.90), index of microcirculatory resistance (iMR),^11^ and coronary flow reserve (CFR).^4^

Taking into consideration the presence of at least moderate stenosis on mid-LAD in a patient with VEBs who wants to practice at least moderate physical activity, to exclude the presence of myocardial ischaemia, invasive coronary angiography (ICA) with full physiology assessment was performed to extensively explore the presence of myocardial ischaemia (both due to epicardial and microcirculation coronary artery disease). Moderate coronary atherosclerosis with 50% obstruction of a lumen in the middle section of LAD was confirmed, and invasive assessment of the functional physiology of the target lesion was also performed with normal values of fractional flow reserve (FFR) (0.90), index of microcirculatory resistance (iMR),^11^ and CFR^4^ (*Figure 2*).

Of note, the high-risk plaque located at the proximal segment of LAD was not well evident at ICA as it was on cardiac CT.

Statin therapy was titrated to atorvastatin 80 mg/die tablets to reach the LDL target of 55 mg/dL, and a low dose of selective beta-blocker, bisoprolol 1.25 mg bid tablets, was started and with indication to maintain a healthy lifestyle with and adherence to the Mediterranean diet; no supplements were prescribed to the patient.

Concerning the eligibility for sports activity, the patient is a master tennis player, the case was discussed with a sport cardiology consultant, and it was deemed appropriate to contraindicate the practice of competitive sports activity with high cardiovascular intensity. On the contrary, non-competitive sports activity with no more than moderate cardiovascular effort, mainly aerobic, was encouraged.

After discharge, the patient was referred for outpatient follow-up with laboratory tests to monitor the lipid and glycaemic profile, Holter EKG during a training session, and stress echocardiography followed by cardiology revaluation after 12 months. The patient is currently in his first year of treatment, and he is asymptomatic and practices without limitation moderate-intensity sports activity, as prescribed. He is proceeding with optimal medical therapy, as prescribed at the time of discharge with LDL cholesterol values at the 12-month check-up still maintaining below the target values (LDL < 55 mg/dL). He did not report any symptoms and was not hospitalized again.

## Discussion

Coronary atherosclerotic disease is the leading cause of cardiovascular death in Western world.

Early CAD identification and plaque analysis by cardiac CT are becoming increasingly valuable in risk stratification for major CV events such as acute myocardial infarction.^11–13^

The use of AI to implement cardiac CT application could make advanced plaque analysis less time-consuming and available even in the absence of an expert cardiac CT reader, potentially helping the referring physicians to routinely obtain detailed non-invasive information on high-risk atherosclerotic disease and set the most appropriate therapy, moving towards precision medicine.^14,15^ Moreover, the use of AI may increase measurement reproducibility which is of utmost importance for monitoring plaque volume and characteristics. However, it should be underlined that potential drawbacks are not negligible in this field in terms of privacy protection, data property, and ethical concerns.

## Conclusion

Cardiac CT nowadays is the most promising tool for non-invasive evaluation of coronary atherosclerotic disease, which has various presentation phenotypes.

Combination with AI could play a leading role in the extensive application of advanced plaque analysis with the potential early identification of high-risk coronary lesions leading to subsequent early aggressive therapeutic management.

## Data Availability

The data underlying this article will be shared on reasonable request to the corresponding author.

## Lead author biography

Dr Edoardo Conte acquired a solid academic and clinical background in the field of cardiovascular imaging, sport cardiology, and myocardial/pericardial disease. He successfully obtained EACVI certification in transthoracic echocardiography, cardiac CT, and cardiac MRI. He is a fellow of the European Society of Cardiology (FESC), a member of the Research Committee of the European Society of Preventive Cardiology (EAPC), and a member of the Myocardial and Pericardial Disease Working Group of the European Society of Cardiology. He is Deputy Editor of the *EHJ-Case Report* and Associate Editor of the *Journal of Cardiovascular Computer Tomography*. He was among the co-authors of 184 research papers.

**Consent:** Informed written patient consent has been obtained, in line with the Committee on Publication Ethics (COPE) best practice guidelines, and the individual who is being reported on is aware of the possible consequences of that reporting.

**Funding:** None declared.

## References

[1] Dalen JE, Alpert JS, Goldberg RJ, Weinstein RS. The epidemic of the 20(th) century: coronary heart disease. Am J Med 2014;127:807–812. 24811552 10.1016/j.amjmed.2014.04.015

[2] Montarello NJ, Nguyen MT, Wong DTL, Nicholls SJ, Psaltis PJ. Inflammation in coronary atherosclerosis and its therapeutic implications. Cardiovasc Drugs Ther 2022;36:347–362. 33170943 10.1007/s10557-020-07106-6

[3] Libby P, Theroux P. Pathophysiology of coronary artery disease. Circulation 2005;111:3481–3488. 15983262 10.1161/CIRCULATIONAHA.105.537878

[4] Hsiao EM, Rybicki FJ, Steigner M. CT coronary angiography: 256-slice and 320-detector row scanners. Curr Cardiol Rep 2010;12:68–75. 20425186 10.1007/s11886-009-0075-z PMC2893879

[5] Nørgaard BL, Jensen JM, Blanke P, Sand NP, Rabbat M, Leipsic J. Coronary CT angiography derived fractional flow reserve: the game changer in noninvasive testing. Curr Cardiol Rep 2017;19:112. 28940026 10.1007/s11886-017-0923-1

[6] Rahoual G, Zeitouni M, Charpentier E, Ritvo PG, Rouanet S, Procopi N, et al Phenotyping coronary plaque by computed tomography in premature coronary artery disease. Eur Heart J Cardiovasc Imaging 2023;13:jead212. 10.1093/ehjci/jead212 37597177

[7] Rodriguez-Granillo GA, Carrascosa P, Bruining N, Waksman R, Garcia-Garcia HM. Defining the non-vulnerable and vulnerable patients with computed tomography coronary angiography: evaluation of atherosclerotic plaque burden and composition. Eur Heart J Cardiovasc Imaging 2016;17:481–491. 26903599 10.1093/ehjci/jew012

[8] Motoyama S, Sarai M, Harigaya H, Anno H, Inoue K, Hara T, et al Computed tomographic angiography characteristics of atherosclerotic plaques subsequently resulting in acute coronary syndrome. J Am Coll Cardiol 2009;54:49–57. 19555840 10.1016/j.jacc.2009.02.068

[9] Salem AM, Davis J, Gopalan D, Rudd JHF, Clarke SC, Schofield PM, et al Characteristics of conventional high-risk coronary plaques and a novel CT defined thin cap fibroatheroma in patients undergoing CCTA with stable chest pain. Clin Imaging 2023;101:69–76. 37311397 10.1016/j.clinimag.2023.06.009

[10] Hansson GK . Inflammation, atherosclerosis, and coronary artery disease. N Engl J Med 2005;352:1685–1695. 15843671 10.1056/NEJMra043430

[11] Conte E, Mushtaq S, Pontone G, Li Piani L, Ravagnani P, Galli S, et al Plaque quantification by coronary computed tomography angiography using intravascular ultrasound as a reference standard: a comparison between standard and last generation computed tomography scanners. Eur Heart J Cardiovasc Imaging 2020;21:191–201. 31093656 10.1093/ehjci/jez089

[12] Andreini D, Magnoni M, Conte E, Masson S, Mushtaq S, Berti S, et al Coronary plaque features on CTA can identify patients at increased risk of cardiovascular events. JACC Cardiovasc Imaging 2020;13:1704–1717. 31422137 10.1016/j.jcmg.2019.06.019

[13] Ahmadi A, Argulian E, Leipsic J, Newby DE, Narula J. From subclinical atherosclerosis to plaque progression and acute coronary events. JACC State-of-the-Art Review. J Am Coll Cardiol 2019;74:1608–1617. 31537271 10.1016/j.jacc.2019.08.012

[14] Jaltotage B, Sukudom S, Ihdayhid AR, Dwivedi G. Enhancing risk stratification on coronary computed tomography angiography: the role of artificial intelligence. Clin Ther 2023;45:1023–1028. 37813776 10.1016/j.clinthera.2023.09.019

[15] Conte E, Annoni A, Pontone G, Mushtaq S, Guglielmo M, Baggiano A, et al Evaluation of coronary plaque characteristics with coronary computed tomography angiography in patients with non-obstructive coronary artery disease: a long-term follow-up study. Eur Heart J Cardiovasc Imaging 2017;18:1170–1178. 27679600 10.1093/ehjci/jew200

